# Periodic Microstructures Fabricated by Laser Interference with Subsequent Etching

**DOI:** 10.3390/nano10071313

**Published:** 2020-07-04

**Authors:** Shuang-Ning Yang, Xue-Qing Liu, Jia-Xin Zheng, Yi-Ming Lu, Bing-Rong Gao

**Affiliations:** State Key Laboratory of Integrated Optoelectronics, College of Electronic Science and Engineering, Jilin University, Changchun 130012, China; yangsn0110@163.com (S.-N.Y.); zhengjx19@163.com (J.-X.Z.); 13180893370@163.com (Y.-M.L.)

**Keywords:** microstructure, GaAs, laser interference, dry etching

## Abstract

Periodic nanostructures have wide applications in micro-optics, bionics, and optoelectronics. Here, a laser interference with subsequent etching technology is proposed to fabricate uniform periodic nanostructures with controllable morphologies and smooth surfaces on hard materials. One-dimensional microgratings with controllable periods (1, 2, and 3 μm) and heights, from dozens to hundreds of nanometers, and high surface smoothness are realized on GaAs by the method. The surface roughness of the periodic microstructures is significantly reduced from 120 nm to 40 nm with a subsequent inductively coupled plasma (ICP) etching. By using laser interference with angle-multiplexed exposures, two-dimensional square- and hexagonal-patterned microstructures are realized on the surface of GaAs. Compared with samples without etching, the diffraction efficiency can be significantly enhanced for samples with dry etching, due to the improvement of surface quality.

## 1. Introduction

In nature, the regular arranged periodic micro/nanostructures on the surface of some organisms endow them with special functions. For example, the micro/nanostructures on the surface of lotus leaves give them the property of self-cleaning [1]; the hybrid periodic micro/nanoscale ripples on the surface of butterfly wings give them a gorgeous schemochrome [2]. Inspired by nature, a variety of periodic micro/nanostructures have been fabricated for applications in anti-reflection devices [3,4], bionics [5,6], invasive regulation [7,8,9], optoelectronic devices [10], organic solar cells [11], and organic light-emitting devices (OLED) [12]. The present processing technologies mainly include plane lithography [13,14], nanoimprinting [15], chemical self-assembly [16,17], and laser direct writing [18,19]. Among these methods, the laser direct writing is useful for the fabrication of complex 3D nanostructures because of its properties of high resolution, controllability, and 3D machining capacity [20,21,22,23]. For example, Chen and coworkers had successfully fabricated hybrid micro/nanostructures with underwater superaerophobic and superaerophilic properties for applications in biomimetics [24]. However, the laser direct writing technology suffers from the time-consuming properties for fabrication of large-area micro/nanostructures. Nevertheless, these methods are unfit for rapid and flexible construction of large-area periodic micro/nanostructures on hard materials with controllable morphologies to satisfy the practical applications. Therefore, a new technology is necessary to rapidly construct periodic micro/nanostructures on hard materials to improve their performance.

Nowadays, laser interference has been demonstrated to be an effective method to fabricate periodic micro/nanostructures [25,26] on various materials, such as polymers [27], ZnS [28], and Si [29]. As for grating structures, there is no need to fabricate them using point-by-point scanning, so it saves the processing time. Laser interference is an inexpensive method since it is mask-free, thus the multi-beam laser interference ablation and interference lithography are used to prepare periodic structures, and some other steps are used if necessary, such as chemical etching [30,31,32]. For processing hard materials, high laser energy is necessary to realize materials’ removal. However, high laser energy will give rise to the increasing of surface roughness of the fabricated periodic micro/nanostructures, which limits their optical applications. Here, we put forward the laser interference with subsequent etching technology to fabricate smooth periodic micro/nanostructures on hard materials. First, two coherent laser beams were used to fabricate periodic structures on the surface of the substrate. Then, the samples were processed by plasma etching technology, including reactive ion etching and ion beam etching. As an example, microgratings with different periods and aspect ratios were demonstrated on gallium arsenide (GaAs). The height of the microstructures were controlled by laser energy and etching time. Due to the improvement of surface smoothness, the diffraction efficiencies of the microstructures were significantly increased after etching. In addition, differently-arranged two-dimensional periodic pyramid microstructures with high diffraction efficiencies were also patterned on GaAs via multiple ablations of two laser beam interferences and subsequent etchings.

## 2. Materials and Methods

**Figure 1:**
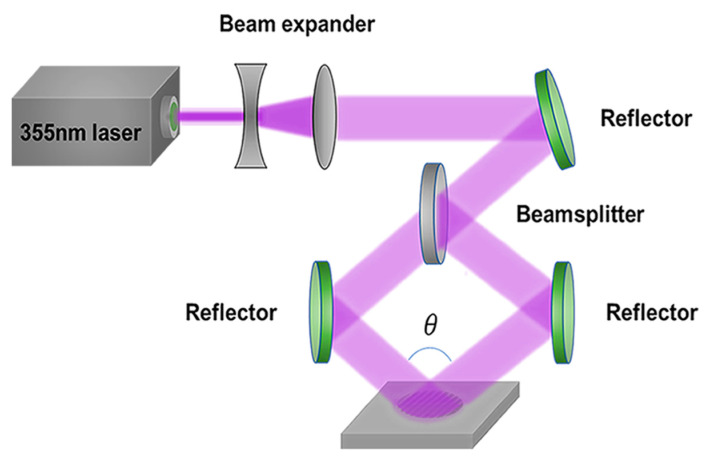
Schematic image of the fabrication of periodic microstructures by laser interference.

Before laser processing, the GaAs substrates were cleaned by an ultrasonic cleaner, including subsequently in acetone and ethanol for approximately 5 min, respectively. Then, the samples were washed by deionized water. The schematic diagram of the two beams of laser interference is shown in Figure 1. By using a beam splitter, a laser beam that was generated by the nanosecond laser (355 nm, 10 Hz, 10 ns) was divided into two coherent lights. Then, the two laser beams converged together to form an interference effect by two reflectors, which directly imprinted on the surface of the GaAs to obtain a micrograting structure with a certain period. The nanosecond laser is a linear polarized laser. The polarization direction is vertical with the plane of the two divided laser beams. After propagating through the beam splitter and the reflector, the polarization direction of the two beams are the same as the initial direction. Although the combination of diffractive optical element (DOE) and two lenses can also be used to fabricate periodic structures with a variety of interference patterns by controlling the polarization direction [33,34], it may cause the damage of DOE by a high power laser, which is necessary to ablate hard materials. Another strategy can also be used to realize laser interference, which consists of asymmetric assigned mirrors [35]. In fact, any laser interference methods that can ablate hard materials are acceptable. Due to the severe thermal effects that were generated in materials by nanosecond laser ablation with high energy, a large number of debris and scattered particles were formed on the sample surface, which affected the field distribution of subsequent laser pulses and resulted in the high surface roughness [9,36]. Here, we proposed post-processing by inductively coupled plasma (ICP, ICP-100A, TAILONG ELECTRONICS, Beijing, China) etching to remove the particles and debris on GaAs. The gas flow rate of Cl_2_ and BCl_3_ were 20 sccm and 5 sccm, respectively. The bias radio frequency (RF) power was 100 W and the antenna RF power was 500 W. The etching time was controlled for different structures.

To measure the surface morphologies of the fabricated microstructures, a field emission scanning electron microscope (SEM, JSM-6700F, JEOL, Tokyo, Japan) was used. The three-dimensional morphologies, cross-section profiles, and surface roughness were measured by confocal laser scanning microscopy (CLSM, OLS4100, OLYMPUS, Tokyo, Japan). The diffraction patterns of different structures on a screen were obtained by a camera.

## 3. Results and Discussion

The period of the microgratings fabricated by two-beam laser interference ablation is in full accord with the distance between maxima and dark sites in the superimposed light field. More materials are removed by laser ablation at the positions with higher light intensity. Therefore, the period can be accurately controlled by laser wavelength (λ) and angle of the two beams (θ) with the following formula [37]:

(1) $$T=\frac{\lambda }{2\sin\frac{\theta }{2}},$$

**Figure 2:**
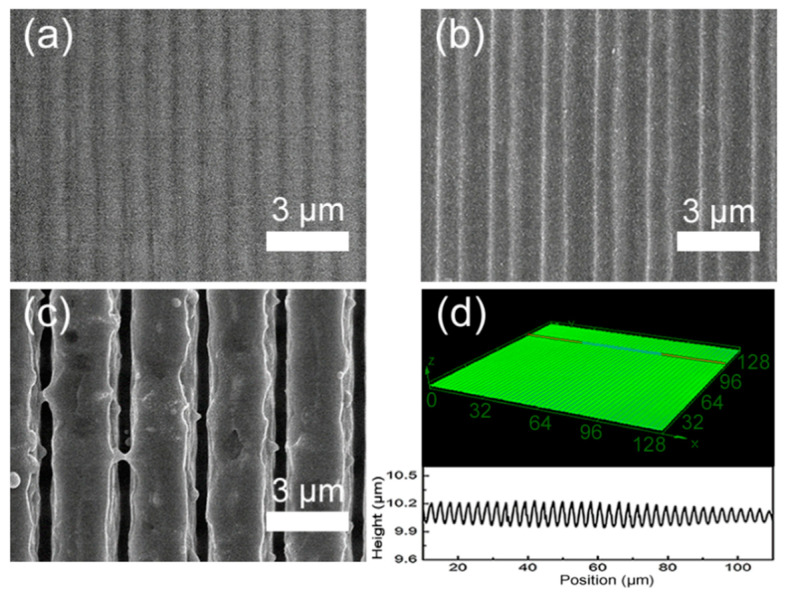
SEM photos of the micrograting with periods of (**a**) 1 μm; (**b**) 2 μm; (**c**) 3 μm; (**d**) three-dimensional topography and cross-section morphology of micrograting with period of 3 μm.

According to the above formula, the period of microstructures can be adjusted by laser wavelength and the angle of two laser beams. Interference phenomena appeared when two beams of light with the same phase and frequency superposed in space, resulting in the redistribution of the light field with alternate formation of maxima and dark stripes [38,39,40]. The coherent light is superimposed at the maxima sites and is weakened at the dark sites. Therefore, more materials are removed at the maxima sites and fewer are removed at the dark ones. A centimeter-sized periodic micrograting can be fabricated by only several pulses according to the laser spot size. Figure 2a–c show the SEM images of interference fringes with periods of 1 μm, 2 μm, and 3 μm by adjusting the angle of two beams, respectively. The laser fluence was 1.2 J/cm^2^ (the spot size was 5 mm^2^ and the laser ablation threshold was about 200 mJ/cm^2^ [41]) and the number of pulses was 20. Thermal diffusion had a great effect on the profile of the microstructures. As the laser interacted with the GaAs, heat flowed from the interference maximum point to the interference minimum point. The material melted when the temperature was high. Due to the long duration of the nanosecond laser pulse, more heat transferred to the interference minimum point, so with the accumulation of heat, the melting of GaAs happened both at maximum and minimum points, which limited the aspect ratio, and since the heat transfer path got longer with the increase of the period, the modulation depth also increased as the heat accumulation at the minimum point decreased (Figure 2a–c). That is to say, under the same conditions, aspect ratio becomes greater as the period gets larger [42]. Due to the severe interaction of the laser pulse and materials, lots of wrinkles and particles were formed on the surface of microstructures, as shown in Figure 2c. The microstructures exhibit a sinusoidal profile and high uniformity, as show in Figure 2d.

**Figure 3:**
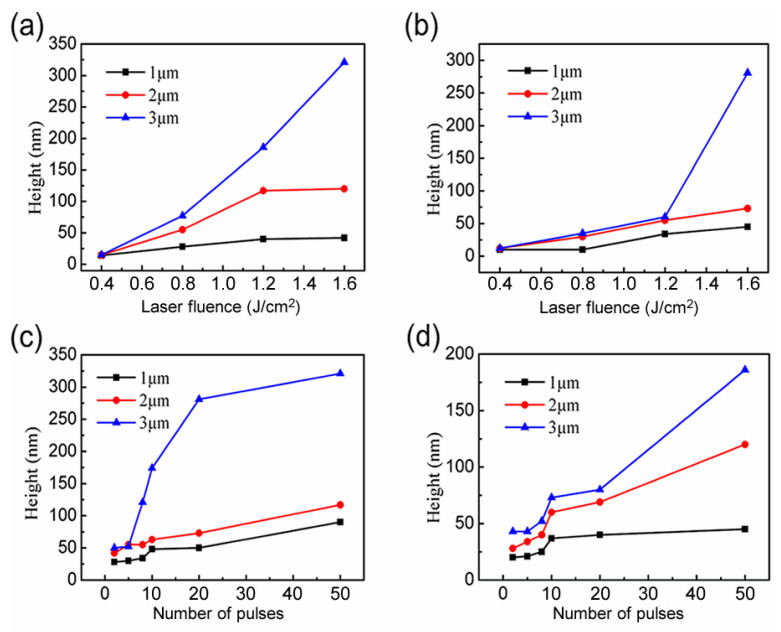
Curves of height variation of different periodic structures. Height varies with the laser power at the exposure time of (**a**) 5 s; (**b**) 2 s; and with the exposure time at a laser power of (**c**) 0.8 W; (**d**) 0.6 W.

The height of a micrograting structure has a great effect on its optical performance. Here, the distribution of the converged optical field determines the height of the microstructure that was fabricated by laser interference. Laser fluence and number of pulses can control the distribution of the converged optical field. Therefore, we investigated the relationship of the height of the microstructure with laser fluence and number of pulses, respectively. In Figure 3a, the number of pulses was fixed to 50 by a mechanical shutter. With the increasing of laser fluence from 0.4 J/cm^2^ to 1.6 J/cm^2^, the height of the microstructure is increased. This is attributed to the increasing of the difference value between the maximum intensity of superimposed light fields and the damage threshold of the substrate, with minimum intensity of superimposed light fields being less than the damage threshold. In the case of the minimum intensity of superimposed light fields being larger than the damage threshold, the height of the microstructure remained unchanged with the increasing of laser fluence. Because the light intensity is larger than the damage threshold in all the superimposed light fields, the height of the microstructure is determined by the difference value between the maximum and minimum intensity of superimposed light fields. The same results can be seen in Figure 3b, where the number of pulses was fixed to 20 and the laser fluence was adjusted from 0.4 J/cm^2^ to 1.6 J/cm^2^. With the increasing of the number of pulses, the laser energy accumulated to the substrate. Therefore, the height of the microstructure was increased with the number of pulses (Figure 3c,d). In addition, more increments of the heights can be observed for microstructures with larger periods in Figure 3. This is due to the different degrees of overlap of the light fields caused by the inconsistent distances between the interference levels of different periods. As the period became larger, the width of the light and dark stripes increased, which means the scope of interaction between the laser and the material expanded, so that there was more material which was removed. The laser fluence and number of pulses increased, which resulted in a more effective interaction with materials. Considering that the thermal diffusion, along with the period, got larger, the thermal modulation depth was greater. So, this was a more pronounced trend.

**Figure 4:**
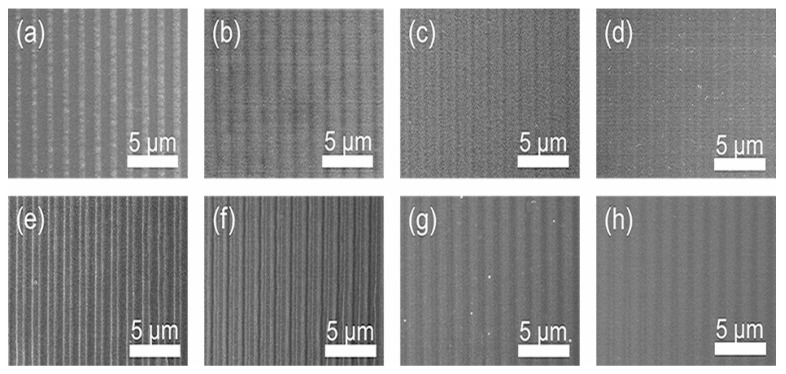
SEM images of microstructures with laser fluence of (**a**) 1.6 J/cm^2^; (**b**) 1.2 J/cm^2^; (**c**) 0.8 J/cm^2^; (**d**) 0.4 J/cm^2^; and number of pulses of (**e**) 50; (**f**) 20; (**g**) 8; (**h**) 5.

Although the height of microstructures can be adjusted by laser fluence and number of pulses, the surface smoothness is not improved. The SEM images in Figure 4a–d exhibit the microstructures fabricated, with the number of pulses of 20 and laser fluence of 1.6, 1.2, 0.8, and 0.4 J/cm^2^, respectively. The substrate was damaged more seriously under higher laser fluence than that under the lower laser fluence, which agreed with the results in Figure 3a,b. In addition, the SEM images in Figure 4e–h show the microstructures fabricated with laser fluence of 1.6 J/cm^2^ and number of pulses of 50, 20, 8, and 5, respectively. The accumulation of laser energy with the increasing of the number of pulses induced more damage to the substrate, which resulted in the increasing of the height and the surface roughness of the microstructures.

**Figure 5:**
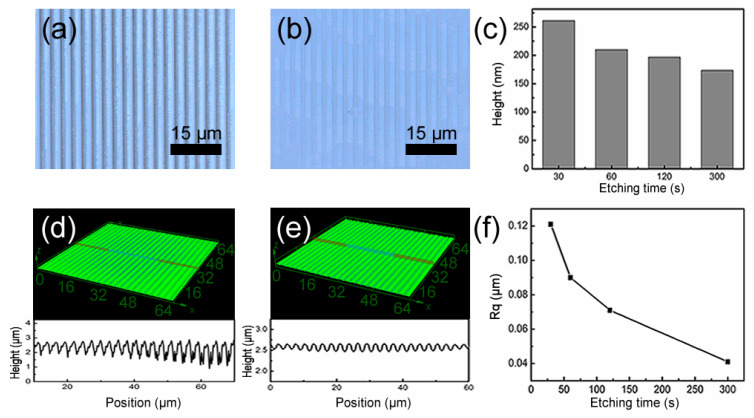
(**a**) Confocal photo before dry etching; (**b**) confocal photo after dry etching; (**c**) histogram of height changes with etching time; (**d**) three-dimensional topography of the microstructure and its cross-section morphology before dry etching; (**e**) three-dimensional topography of the microstructure and its cross-section morphology after dry etching; (**f**) the variation of surface roughness with etching time.

For an optical device, the surface roughness is a very important parameter that influences the optical performance. With high surface energy, the wrinkles and particles that are induced by laser ablation can be rapidly removed by dry etching [43,44]. So, the dry etching process was introduced to reduce the surface roughness of the microstructures that were fabricated by laser interference. Figure 5a,b show the morphology of microstructures with the period of 3 μm before and after etching, respectively. It was found that the debris on the surface of microstructures decreased after etching, which was demonstrated by the comparison of the cross-section profile and three-dimensional topography of the structures before and after etching, as shown in Figure 5d,e. Because of the insufficient amount of etching gas on the bottom of the gratings, the height of the microstructures gradually decreased with the increasing of the etching time (Figure 5c). Thus, the height of the microstructures was also controlled by the etching time to satisfy the special applications. In addition, the surface roughness of the structures also decreased with the increasing of the etching time (Figure 5f). For example, the surface roughness of the microstructures was decreased from 140 nm to 40 nm with the etching time increasing to 5 min.

**Figure 6:**
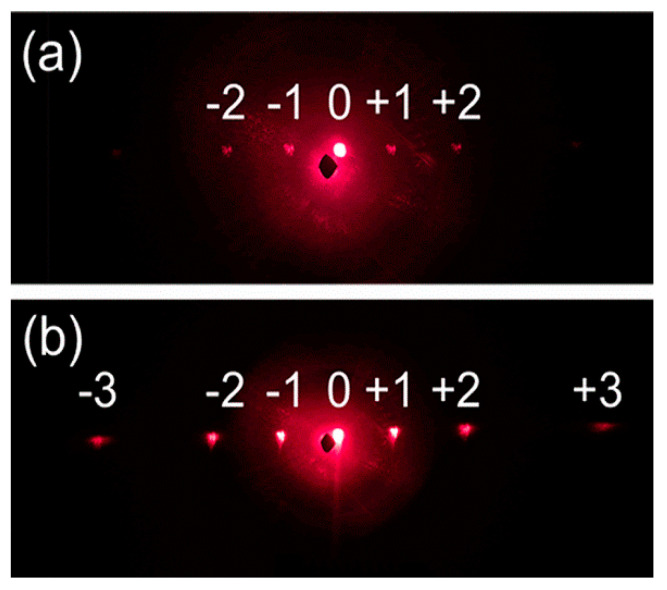
(**a**) Diffraction pattern of the nanograting structure before etching; (**b**) diffraction pattern of the grating structure after etching.

The diffraction efficiency of the microgratings were affected by the surface roughness. As the surface quality of the etched samples was greatly improved, the diffraction efficiency was also improved. To measure the diffraction property, a continuous laser with a wavelength of 633 nm was irradiated to the surface of the GaAs, and the diffracted light spot was obtained by a camera on a screen after reflection. Figure 6a,b show the optical images of diffracted light patterns that were obtained from microstructures without and with etching, respectively.

Except for the zero-order and the first-order diffraction spots, only the second-order diffraction spot was distinguished from the screen by the microstructures fabricated before etching (Figure 6a). However, the third-order diffraction spot was also distinguished by the microstructures fabricated after etching. In addition, the brightness of the first-order and second-order diffraction spots were significantly enhanced, which were attributed to the improvement of the surface smoothness.

**Figure 7:**
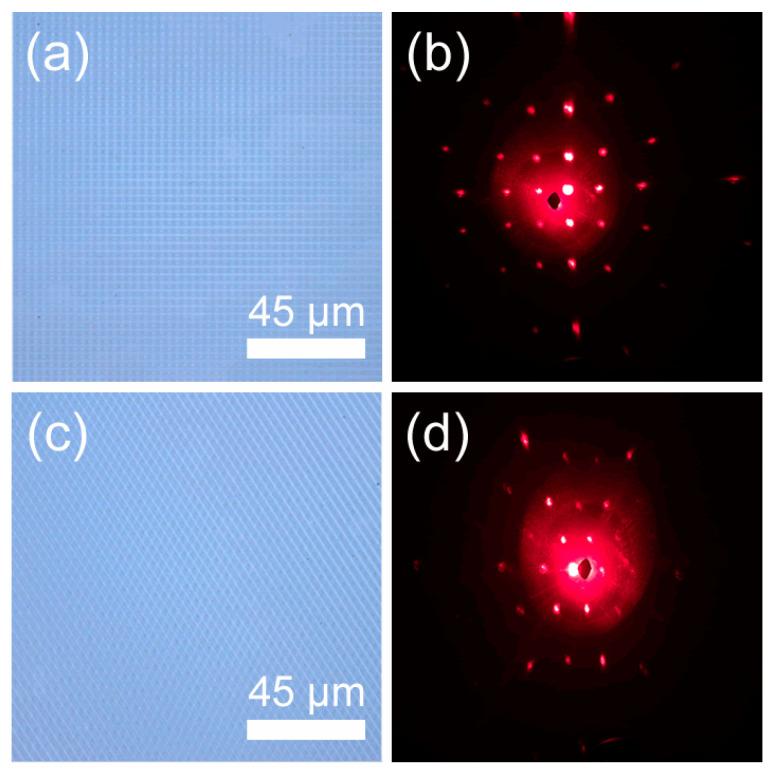
(**a**) Confocal photograph of square pattern; (**b**) diffraction pattern of square pattern; (**c**) confocal photograph of hexagonal pattern; (**d**) fiffraction pattern of hexagonal pattern.

Except for microgratings, a two-dimensional square pattern and hexagonal pattern was also fabricated by the two-beam interference with angle-multiplexed exposures [28]. First, the grating structure was prepared by a single exposure, and then the sample was rotated for multiple exposures. In the experiment, we demonstrated the fabrication of a two-dimensional square pattern and hexagonal pattern on the GaAs by laser interference with the subsequent etching method. The square pattern was fabricated by applying a second exposure by rotating the sample by 90 degrees. Similarly, the hexagonal pattern was fabricated by superimposing coherent light twice by rotating the sample by 60 degrees. It is worth mentioning that in the process of multiple exposures, the number of pulses required for the second and the third exposure should be reduced, which was determined by the modification to the materials by laser. The damage threshold of the materials was reduced after laser modification. So, the number of pulses required for the second and third exposures lessened. Figure 7 shows the optical images and diffraction patterns of the square- and hexagonal-patterned structures, respectively.

As the photos in Figure 7a,c show, the structures are uniform and consistent with the expected morphology. Meanwhile, the period is roughly the same as the design, and the diffraction patterns also completely conform to the structure’s arrangement, showing a square and a hexagonal shape (Figure 7b,d). It is indicated that a variety of micro/nanostructures can be fabricated by two-beam interference and has good application prospects.

## 4. Conclusions

In summary, a laser interference with subsequent etching technology is proposed for efficient preparing of periodic micro/nanostructures with large area and high surface quality. One-dimensional microgratings and two-dimensional square- and hexagonal-patterned microstructures were realized on GaAs with this technology. The height of the periodic microstructures were controlled by laser fluence, the number of pulses, and etching time. In addition, the surface roughness was significantly reduced by the subsequent dry etching process. Due to the improvement of the surface quality, the third-order diffraction patterns were clearly distinguished on the screen via the microstructures fabricated after etching, which was not seen for the samples without the etching process. Therefore, the technology has potential applications in flexible fabrication of uniform periodic micro/nanostructures on hard materials, with good optical performance.
